# Worm Phenotype Ontology: Integrating phenotype data within and beyond the *C. elegans *community

**DOI:** 10.1186/1471-2105-12-32

**Published:** 2011-01-24

**Authors:** Gary Schindelman, Jolene S Fernandes, Carol A Bastiani, Karen Yook, Paul W Sternberg

**Affiliations:** 1 Howard Hughes Medical Institute, California Institute of Technology, Pasadena, CA 91125, USA; 2 Division of Biology, California Institute of Technology, Pasadena, CA 91125, USA

## Abstract

**Background:**

*Caenorhabditis elegans *gene-based phenotype information dates back to the 1970's, beginning with Sydney Brenner and the characterization of behavioral and morphological mutant alleles via classical genetics in order to understand nervous system function. Since then *C. elegans *has become an important genetic model system for the study of basic biological and biomedical principles, largely through the use of phenotype analysis. Because of the growth of *C. elegans *as a genetically tractable model organism and the development of large-scale analyses, there has been a significant increase of phenotype data that needs to be managed and made accessible to the research community. To do so, a standardized vocabulary is necessary to integrate phenotype data from diverse sources, permit integration with other data types and render the data in a computable form.

**Results:**

We describe a hierarchically structured, controlled vocabulary of terms that can be used to standardize phenotype descriptions in *C. elegans*, namely the Worm Phenotype Ontology (WPO). The WPO is currently comprised of 1,880 phenotype terms, 74% of which have been used in the annotation of phenotypes associated with greater than 18,000 *C. elegans *genes. The scope of the WPO is not exclusively limited to *C. elegans *biology, rather it is devised to also incorporate phenotypes observed in related nematode species. We have enriched the value of the WPO by integrating it with other ontologies, thereby increasing the accessibility of worm phenotypes to non-nematode biologists. We are actively developing the WPO to continue to fulfill the evolving needs of the scientific community and hope to engage researchers in this crucial endeavor.

**Conclusions:**

We provide a phenotype ontology (WPO) that will help to facilitate data retrieval, and cross-species comparisons within the nematode community. In the larger scientific community, the WPO will permit data integration, and interoperability across the different Model Organism Databases (MODs) and other biological databases. This standardized phenotype ontology will therefore allow for more complex data queries and enhance bioinformatic analyses.

## Background

Phenotypes are the observable physical or biochemical traits manifested by an organism in response to their genetics and environment. Phenotype designation has long been the mainstay for geneticists, allowing scientists to infer gene function from the phenotypes and genetic properties of mutations [1-3]. As methods for analyzing gene function continue to evolve, identifying and characterizing phenotypes necessarily requires a means to organize phenotype information into a unified vocabulary that will allow researchers to realize that seemingly disparate gene activities may actually be affecting a similar biological process. With the publication of an essentially complete genome sequence in 1998 [4] and the definition of a complete gap-free sequence in 2005 [5], virtually every gene in *C. elegans *became accessible to functional analysis based on phenotypes via reverse genetics [6]. As a consequence, information from classical genetics is now complemented by high-throughput RNAi screens, individual RNAi experiments, and gene knockout data [7-9]. It has been estimated that 79% of *C. elegans *protein-coding genes have a known protein motif, and 21% of them have non-nematode orthologs [10]; hence, the ability to functionally and molecularly characterize these protein products is of profound consequence to the scientific field outside of the *C. elegans *community.

*C. elegans *is amenable to various other methods of genetic or physical manipulation, and the phenotypic outcomes of these modifications are also useful to discern gene function. These manipulations include transgene overexpression, cell ablation, pharmacological treatment, and genetic mosaic analysis. In addition, the phenotypic consequence of multiple modifications within the same strain describes a genetic interaction, instrumental in defining whether genes act in parallel or intersecting genetic pathways [11].

WormBase (http://www.wormbase.org) serves as a repository for the wealth of phenotype data in *C. elegans. *The primary literature serves as the main source for phenotype data, but WormBase also receives information from individual researchers and gene knockout consortiums through direct submissions to the database [8,9]. The vocabulary used to describe similar or identical phenotypes, as well as the level of descriptive resolution, often varies between these sources. Prior to July 2006, mutant alleles in WormBase (version WS160) were annotated using a free-text format. Consequently, different words were used to annotate the same phenotypes and there was no inherent hierarchical organization in the descriptions, thus making retrieval of phenotypic information via searches more cumbersome. Perhaps more importantly, although free-text data is accessible by humans, it is not readily available in a computable form and therefore hinders the ability to perform standard bioinformatic operations such as term enrichment analyses or the clustering of annotations. Lack of structure also makes it difficult and time consuming to draw effective comparisons within and between different organisms.

As a step towards overcoming these challenges, we initially developed a phenotype vocabulary limited to 127 phenotype terms in WormBase. These terms were mainly the 3-letter phenotype descriptors familiar among *C. elegans *researchers; such as 'Dpy' (dumpy), 'Unc' (uncoordinated), and 'Bli' (blistered). This vocabulary did not contain phenotype term definitions or references, and there was redundancy within these terms (for example both 'Prz' and 'Prl' stood for paralyzed). We developed this initial vocabulary mainly to accommodate the rapidly ballooning influx of phenotype data from large-scale RNAi experiments, and continued to use free-text descriptions for alleles. Compared to phenotypes that describe the outcome of RNAi analyses, mutant allele descriptions are typically more variable, as they have accumulated over a longer period of time, tend to be more granular (specific), and arise from many independent studies. We quickly realized that to integrate the massive amount of phenotype data across different sources, permit integration with other data types, and render the data computationally accessible, a controlled vocabulary unifying identical, similar, and related concepts was essential for optimal synthesis of accumulated data within and beyond the *C. elegans *research community.

With the above objectives in mind, we built the Worm Phenotype Ontology (WPO) to organize and classify phenotype data in *C. elegans*, utilizing ontology structure and rules set up by some of the other model organism databases (MODs) [12-14]. An ontology is a controlled vocabulary organized in a hierarchical-structure intended to represent relationships between human-interpretable concepts. Moreover, because an ontology uses a controlled vocabulary with strict relationships between its terms, it is computer-comprehensible and thus allows for complex data queries [15]. Ontologies have also proven to be powerful tools for curation consistency, as well as cross-species comparison of biological data [12-14,16]. Perhaps the best example of how the use of ontologies has aided in the dissemination and integration of research data across many fields is found in the widespread use of the Gene Ontology (GO) [16].

Many organism-specific ontologies are however primarily designed to cater to the needs of their individual user communities and are not mutually interoperable. The absence of cross-operability makes it extremely challenging for users to merge existing genotype-phenotype annotations from the different organism databases and compare data across species. Integration of phenotype data thus depends on the existence of cross-products with other ontologies, a goal that is facilitated by the PATO project at the OBO Foundry (http://obofoundry.org/wiki/index.php/PATO:About). PATO-facilitated cross-products enable approximate equivalence mappings with independent phenotype ontologies developed for different organisms [17]. For example, the Mammalian Phenotype Ontology term 'Spherocytosis' can be expressed as being equivalent to the cross-products of the terms 'Erythrocyte' and 'Spherical' from the OBO Cell ontology and PATO, respectively [17]. The creation of equivalence mappings is therefore a way of linking concepts from different ontologies so users of a particular ontology can access comparative information from other ontologies.

Although the Worm Phenotype Ontology (WPO) was initially aimed at the curation of *C. elegans *phenotypes, we have extended its application to include phenotypic data from other nematode species. This expansion mirrors the recent evolution of WormBase to include the complete genomic sequence, gene predictions and orthology assignments from a range of nematodes [18]. Furthermore, we have increased the comparative value of the WPO by generating equivalence mappings for individual process-oriented phenotype terms to GO terms, thereby promoting cross-operability across different biological databases. Therefore, the organized framework of the WPO will provide non-nematode biologists with a means to interact with WormBase curated data. Finally, a structured machine readable vocabulary will allow complex data queries, expediting the identification of genes that act in the same processes or pathways, ultimately across organisms, and thus conserve valuable researcher time, effort and resources.

## Results

### Structure of the WPO

The overall hierarchy of WormBase's phenotype ontology mirrors the rationale and organization employed by the Gene Ontology (GO) consortium (http://www.geneontology.org) as a directed acyclic graph (DAG). Terms represent phenotype classes, and a child term represents a subclass of its parent term. Child terms in the phenotype ontology all hold an 'is_a' relationship with their parent terms. The is_a relationship is transitive, implying that if 'Phenotype A' is_a 'Phenotype B', and 'Phenotype B' is_a 'Phenotype C', then 'Phenotype A' is_a 'Phenotype C.' The hierarchical structure thus allows phenotype annotation to be made at a granular level while preserving the association of child terms to a more general parent term.

**Figure 1:**
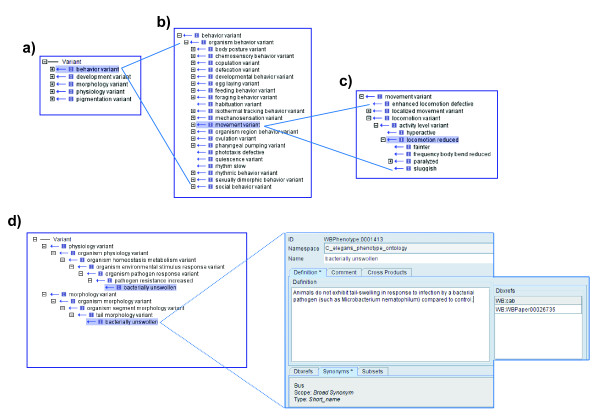
**The hierarchical structure of the WPO**. **(a) **The five children of the root term 'Variant' as viewed in OBO-Edit [63], the ontology editing tool in use at WormBase. **(b) **Each of these five terms (classes) has multiple descendants, as illustrated by the children and grandchildren of the 'behavior variant' term. The '+' sign in the box denotes that descendent terms are present. Clicking on the "+" sign in OBO-edit reveals the subclasses. The lowercase 'i' icon denotes the 'is_a' parent-child relationship between terms. **(c) **Under 'movement variant', 'locomotion reduced' is a visible subclass. Among its descendants are 'paralyzed' and 'sluggish' (see text for details). **(d) **On the right is the OBO-Edit display of the 'bacterially unswollen' phenotype class including a unique identifier (ID), primary name (name) and the definition of the term with references (Dbxrefs i.e., database references). The references in this case are a specific WormBase curator (cab is Carol A Bastiani) and a paper reference [65]. Below the definition are synonyms for this term. In this case, 'Bus' is a three-letter synonym familiar to the *C. elegans *community. On the left is the placement of 'Bus' in the WPO. Note it has two parents, 'pathogen resistance increased' and 'tail morphology variant'.

The Worm Phenotype Ontology contains one root term, 'Variant', with five direct descendants (children) (see Figure 1a). The root term, 'Variant', reflects the fact that the "control" animal for an experiment is defined as a reference strain, with characteristic properties. A phenotype that differs from the phenotype of the reference strain is defined as 'Variant.' The five direct children of 'Variant' encompass the major classes of phenotypes in *C. elegans *and other nematodes: 'behavior variant', 'development variant', 'morphology variant', 'physiology variant' and 'pigmentation variant'. As shown in Figure 1a, the relationship between 'Variant' and 'behavior variant' is an is_a relationship where 'Variant' is the parent and 'behavior variant' is the child. Therefore the children of 'behavior variant', such as 'organism behavior variant' are grandchildren of 'Variant' (see Figure 1b).

A phenotype term (also referred to as a primary name or name) is assigned a unique 'WBPhenotype' identifier; for example, 'bacterially unswollen' is WBPhenotype:00001413. Each phenotype term is also associated with a definition, references and synonyms (where appropriate). Synonyms are assigned so as to allow for non-uniform community jargon to denote the same type of entity without compromising efficacy and accuracy of term nomenclature. References can include the primary literature, GO term definitions or WormBase curators (see Figure 1d). To researchers both inside and outside the *C. elegans *community the term 'bacterially unswollen' may not be familiar; however, the placement of the terms in the ontology reflects the biology. The organization is such that a non-*C. elegans *researcher can browse the ontology without any prior knowledge of *C. elegans *biology or jargon. For example, the 'bacterially unswollen' phenotype, which describes worms that do not exhibit a tail-swelling response to infection by a bacterial pathogen, is a descendant in both the 'physiology variant' and 'morphology variant' branches. In terms of physiology, it is related to pathogen resistance; in terms of morphology it is related to tail morphology (see Figure 1d). The DAG structure permits individual terms to be children of multiple, broader parent terms.

**Figure 2:**
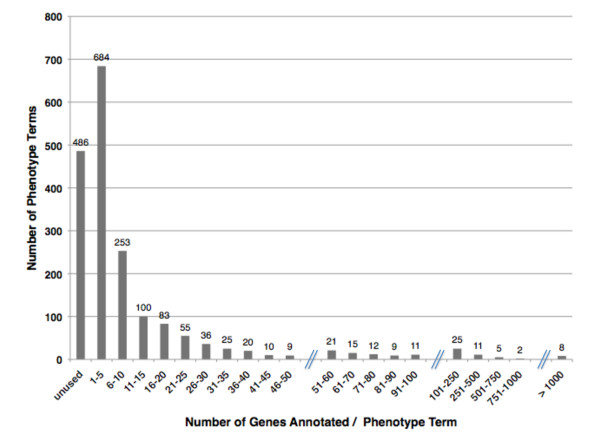
**WPO term usage**. Shown is the distribution of the number of phenotypes (y-axis) with the indicated number of genes annotated per phenotype term (x-axis). Of the 1880 phenotype terms in the WPO, 486 (26%) are unused. Of the remaining terms, 684 have been used to annotate between 1 to 5 genes. 253 terms have been used to annotate between 6 and 10 genes, and so on. The most used phenotype term is 'embryonic lethal', which has been used to annotate 3304 genes (not shown, 'embryonic lethal' is one of 8 terms that have been used to annotate greater than 1000 genes).

There are now a total of 1880 phenotype terms in the WPO, all of which are defined along with their respective references. 74% of the terms in the ontology are used in annotation. Of the remaining 26% of unused terms, many of these provide structure for more granular terms that were considered necessary to capture the appropriate level of detail for a phenotype annotation so as to reflect its description in the literature. Unused phenotype terms are expected to be used as phenotype annotations in WormBase continue to grow. The current usage of phenotype terms annotated to genes is summarized in Figure 2. 12% of terms have two or more parents (number of terms with multiple paths) as multiple inheritance relationships allow us to capture the different biological contexts of a phenotype within the overall hierarchical structure [19] (See the 'bacterially unswollen' example in Figure 1d). The WPO is in compliance with The OBO Foundry principles (http://www.obofoundry.org/crit.shtml), making it a ready source for ontology users from other fields. Currently, The Biological General Repository for Interaction Datasets (BioGRID) database is utilizing the WPO for the annotation of phenotypes related to genetic interactions in *C. elegans *[20].

### Development of the WPO

Initially we evaluated the preexisting 127 phenotype classes in WormBase and placed them within the five major classes of phenotypes in the WPO. Related terms were relegated to an appropriate location within the same branch of the hierarchy. For example, 'paralyzed' and 'sluggish' ('Slu') are both descendants of the 'locomotion reduced' branch of the ontology found in the 'behavior variant' section (see Figure 1c). Also, terms judged to be equivalent were merged (for example 'Prl' and 'Prz' for 'paralyzed'). When terms were merged, original names were maintained as synonyms, and synonyms were linked to relevant papers or curators so that the source for the names can be tracked.

**Figure 3:**
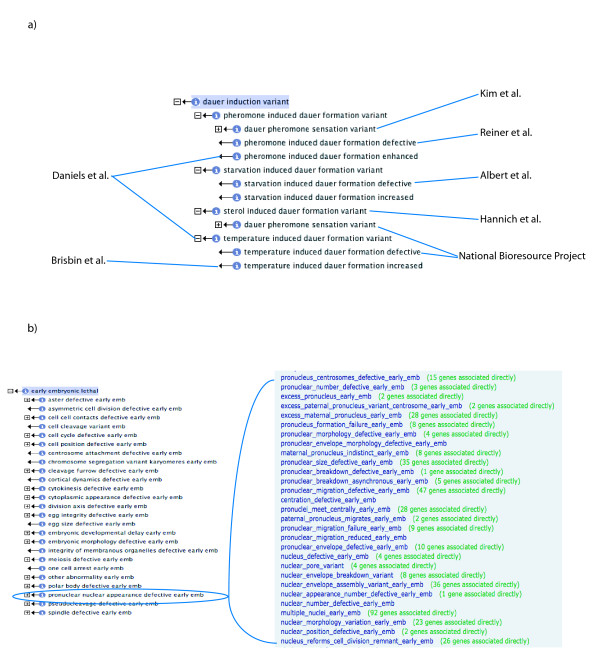
**Forces driving the development of the WPO**. Curation of *C. elegans *literature helps to increase the robustness of the phenotype ontology and we create terms as needed. Ontology views within OBO-Edit. **(a) **Blue lines point to the reference for the term. In some cases more than one term is created from a single reference [9,66-71]. **(b) **Expert input leads to extensive granularity in the ontology. There are 29 descendants of the 'pronuclear nuclear appearance defective early emb' branch (bracketed box), which was refined by soliciting feedback from the community.

The development of the WPO has been driven by curation of the primary literature; specifically, we prioritize the creation of terms based on need. One example of how a branch of the ontology becomes more refined through curation can be found in the 'dauer induction variant' branch (see Figure 3a). The earlier versions of the WPO lacked child terms for 'dauer induction variant', yet there are multiple ways to induce dauer formation in *C. elegans*: starvation, temperature change, pheromone application and sterol application. As we read papers pertaining to dauer biology this branch of the ontology became more refined. In addition to the papers we actively curate we also use Textpresso (http://www.textpresso.org, an information extraction tool for biological literature) [21], WormAtlas (http://www.wormatlas.org, a site dedicated to worm anatomy), and WormBook (http://www.wormbook.org, a source of biological process reviews) [22] to help create and refine terms, definitions and synonyms. As the WPO becomes more encompassing for a particular biological phenomenon terms are added less frequently.

We also actively solicit expert input from members of the research community to create and define terms and to ensure that the ontology reflects the biology of *C. elegans*. Terms that describe early embryonic lethal phenotypes ('early emb') were developed in this fashion. As a result, embryonic lethal descendent terms represent the most robust, and intricate portion of the WPO (see Figure 3b). There are 116 embryonic lethal terms, developed initially by integrating and ordering phenotypes described in publications where the researchers performed high-throughput RNAi screens [23-27]. Extensive development of this branch reflects our general policy of ontology development, which is to mirror the research direction of the community, and is influenced by the particular technological advantages offered by *C. elegans *as a model system, in this case RNAi.

### Expanding the scope and utility of the WPO

Initially our ontology used 'Abnormal' as the root term, instead of 'Variant', and reflected the fact that "wild type" is defined as a genetically homogenous reference strain, Bristol N2, and for practical purposes, accepted as such by the *C. elegans *community. Recently, other nematodes besides *C. elegans *var. Bristol N2 have been incorporated into WormBase including five additional *Caenorhabditis *species (*C. briggsae*, *C. remanei*, *C. brenneri*, *C. japonica *and *C*. sp. 3 PS1010) and four non-*Caenorhabditis *nematodes (*B. malayi, M. incognita, M. hapla and P. pacificus) *[18]. However, the use of 'Abnormal' in WPO term names would preclude the annotation of phenotypes to these species. Moreover, the 'Abnormal' qualifier also prevents the annotation of closely-related *C. elegans *natural variants (e.g., *C. elegans *var. Hawaii) as characteristics vary among strains/isolates of the same species, but are not deemed abnormal. An example of this is the deposition of a mating plug after copulation. *C. elegans *var. Bristol N2 does not deposit a mating plug after copulation, however *C. elegans *var. Hawaii does [28]. Therefore we chose to use 'Variant' as the root term to avoid comparing everything to Bristol N2.

In addition to replacing 'Abnormal' with 'Variant' as the root term, our phenotype ontology underwent a number of revisions with respect to term definitions so as to include non-hermaphroditic species. For example, the term 'egg morphology abnormal', was changed to 'egg morphology variant' and defined as, "Any variation in the overall structure or appearance of fertilized oocytes that are laid compared to those laid by control animals." Because we refer to control and not "wild type" or "N2", we are no longer limited to applying this term to a hermaphroditic species. Furthermore, the term can be used regardless of egg morphology differences among species, as the control is the reference strain. Phenotype terms still exist within the ontology to describe alterations in hermaphrodite-specific phenomena and new terms can be created to accommodate female and male specific terms from other nematode species. When we made this switch we retained the 'Abnormal' version of the term as a synonym so users accustomed to the previous nomenclature could still find terms of interest.

### Phenotype assignments to non-*C. elegans *nematodes

**Figure 4:**
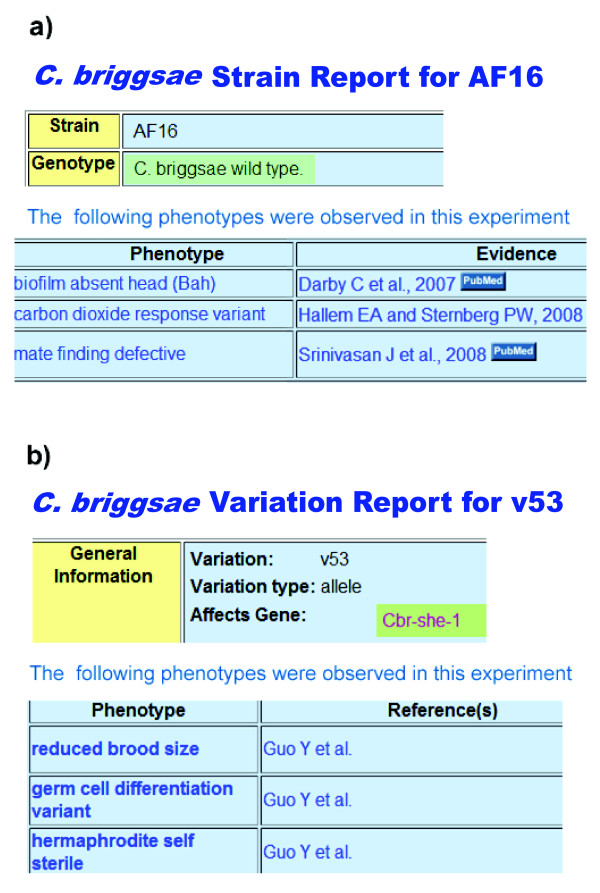
***C. briggsae *phenotype assignments in WormBase**. *C. briggsae *is a nematode species that is closely related to *C. elegans *[72]. **(a) **Shown are excerpts from the AF16 strain page, a wild-type form of *C. briggsae*, which reports the associated phenotype annotations and the corresponding references that describe the controls for each of the experiments. **(b) **Shown are excerpts from the *v53 *variation report page, listing observed phenotypes and corresponding references. *v53 *is a *C. briggsae she-1 *mutant.

As mentioned above, precluding the use of Bristol N2-specific phenotype terminology enables us to curate natural variants (e.g., Hawaii) and assign accurate and consistent phenotypes to non-Bristol N2 strains and mutants (Figure 4). The ability to query genomic databases via a standardized phenotype ontology used across species will facilitate the linking of evolutionary outcomes across those species with pertinent genetic changes, providing evolutionary biologists with a foundation for dissecting fundamental pathways and processes. For instance, even though both *C. elegans *and *C. remanei *descended from a gonochoristic ancestor, *C. elegans *exhibits hermaphroditism [29]. Recently, it has been argued that the evolution of hermaphroditism in *Caenorhabditis *can be attributed to a *tra-2 *mutation in the sex-determination pathway that causes *XX *spermatogenesis and a *swm-1 *mutation that allows these spermatids to self-activate [30]. Therefore, retrieving genes annotated to 'spermatogenesis variant' in these non-*C. elegans *species can provide a vocabulary useful to evolutionary biologists that choose to dissect these pathways.

### Annotating allele, RNAi and transgene overexpression phenotypes in *C. elegans*

**Figure 5:**
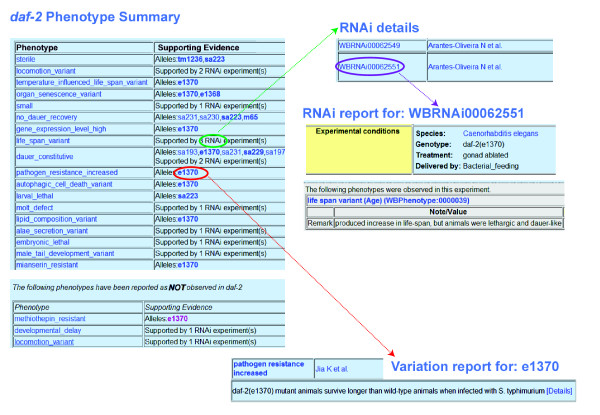
***C. elegans *phenotype assignments in WormBase**. Shown are excerpts from the *daf-2 *gene page in WormBase. Phenotypes associated with alleles, RNAi experiments or transgenes (not shown) can be viewed in the phenotype summary tables. The *e1370 *allele object has its own specialized 'Variation Report' page that can be accessed through links, marked with a red oval, embedded in the phenotype summary tables on the Gene Summary page. The phenotype summary tables include a list of phenotypes associated with knockdown via RNAi for *daf-2 *(green oval). A more detailed overview of this RNAi experiment can be found within the 'RNAi details' section. The details section also contains links to a specific experiment, called the 'RNAi Report', via the WBRNAi ID (purple oval). The phenotype summary also includes 'Not' phenotype annotations (bottom left).

The utility of the Worm Phenotype Ontology is especially apparent when browsing Gene Summary pages, Variation Reports, Transgene Summary pages, and RNAi Reports at the WormBase site (http://www.wormbase.org/). The main portal for access to phenotypic data is through the WormBase Gene Summary page (Figure 5). The phenotype summary tables in the 'Function' section of the Gene Summary page includes a list of phenotype associations to a specific gene, as well as a list of phenotypes specifically reported as not being associated with that gene. Phenotypes not associated with a gene are prefaced by a 'Not' qualifier. This usage means researchers looked for this specific phenotype, but did not observe it in the reported experiment. For example, in Figure 5, the phenotypes 'methiothepin resistant', 'developmental delay' and 'locomotion variant' were not observed when the *daf-2 *gene was disrupted. The use of the 'Not' qualifier eliminates the need to duplicate every term in the WPO in the negative. A summary of the current nembers to allele, RNAi and transgene overexpression phenotypes are shown in Table 1.

**Table 1:** Annotation summary^a^

Data Object	Number of Genes Annotated	Number of Phenotype Connections^b^
**Alleles**	4361**^c^**	19824
**RNAi**	18834^d^	297909
**Transgenes (Overexpression)**	109^e^	461

^a ^Data from the WormBase WS215 release.

^b ^Includes both observed and 'Not' observed phenotypes. The number of phenotype connections is greater than the number of genes annotated, as each gene may have multiple phenotypes associated with them.

^c ^Derived from 7066 alleles.

^d ^Derived from 79068 RNAi experiments.

^e ^Associated with 223 transgenes.

### Data mining and searches

**Figure 6:**
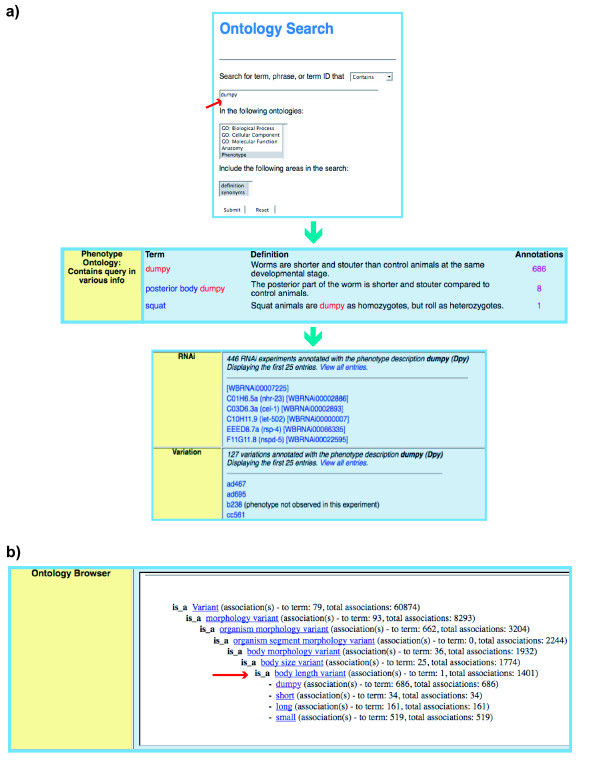
**Data mining using the ontology search tool in WormBase**. **(a) **A user may enter a query term in the search box; in this case 'dumpy' is used as an example. Results displayed in the output include the terms that contain 'dumpy' within the term name or within its definition (highlighted in red). Clicking on the number to the right, which indicates the total number of annotations to each term, retrieves RNAi, allele (variation) and transgene objects associated with a phenotype. Displayed is a portion of the 686 annotations made to 'dumpy'. There are RNAi and variation objects associated with this term, but no transgene data. **(b) **Included in the ontology search output (shown here for 'dumpy') is a window that allows the user to browse the ontology. If a user clicks on a term, the children of that term are revealed as well as the number of genes associated to that term. Shown is one gene directly annotated to 'body length variant' (red arrow), but 1401 total associations are indicated, as this number includes all the annotations to the children ('dumpy', 'short', 'long' and 'small').

WormBase has implemented a phenotype ontology search tool to integrate experimental phenotype data from RNAi experiments, alleles, and transgenes. Using the ontology search (found on the main WormBase page under searches) a user can input a term, phrase, synonym (e.g., Dpy) or ID to search for annotations connected to that term. For example if a user enters 'dumpy' in the search field (see Figure 6a) and selects the phenotype ontology, by default the search will look for 'dumpy' in the term name, definition and synonyms fields. The output (see Figure 6a) shows term names that contain 'dumpy' and terms that use 'dumpy' in their definitions. Each term shown in the browser is followed by a hypertext link listing the number of annotations in WormBase to each term and/or to children of that term (see Figure 6a). In addition, on the phenotype term pages in WormBase one can browse the term names in the ontology (see Figure 6b). Thus, when coupled with the new ontology search tool, the ontology's controlled vocabulary facilitates the retrieval of allele, RNAi, transgene, or strain objects that have equivalent phenotypes. In addition, the organization of the ontology facilitates the retrieval of objects annotated to phenotypes that are considered to be a more defined subclass of a phenotype term. For example one might want all the annotations to 'locomotion reduced', which would include 'fainter', 'paralyzed', etc., or one might just want the annotations that are directly associated with one of these terms (see Figure 1c).

In addition, to facilitate phenotype term enrichment analysis, the WPO contains a "WPO slim". A slim is a streamlined version of the ontology that contains a subset of the terms in the whole WPO. This subset is meant to give a broad overview of the ontology content without the details of the more specific granular terms.

### Using PATO-GO cross-products to integrate the WPO with other organism databases

Phenotypes are typically described by using either a species-centric pre-composed ontology or using a more general post-compositional approach, drawing from various ontologies [31-35]. In a pre-composed ontology, phenotype terms are already defined and placed within the hierarchical structure of the ontology, such as the WPO. To describe a phenotype term using a post-compositional approach, a bipartite "EQ" (Entity + Quality) schema is employed and the entity of interest is described by a quality [36]. For example, in the case of the phenotype 'shrunken intestine', the entity is 'intestine' (WBbt:0005772) and the quality is 'shrunken' (PATO:0000585) [17]. The quality terms are derived from the Phenotype and Trait Ontology (PATO) [37], which can be used in conjunction with species-specific anatomical ontologies or cross-species entity ontologies [38,39]. This flexible post-compositional approach has already been employed in the annotation of human genotype-phenotype associations, as well as in model organism databases such as FlyBase (*Drosophila*) and ZFIN (zebrafish) [31,34].

We used a pre-composed approach to create the WPO because pre-composed ontologies are able to incorporate community specific jargon, some of which is not amenable to the EQ schema (because it fails to capture the term's biological complexity). The 'kinker' phenotype is one case in which non-*C. elegans *users would not intuitively grasp its relationship to locomotory behavior without the benefit of a pre-coordinated hierarchy. Although our pre-composed ontology serves the data mining needs of nematode researchers, its lack of cross-operability would have the effect of rendering worm phenotype terms as opaque and less accessible to researchers using other organisms in their research.

**Figure 7:**
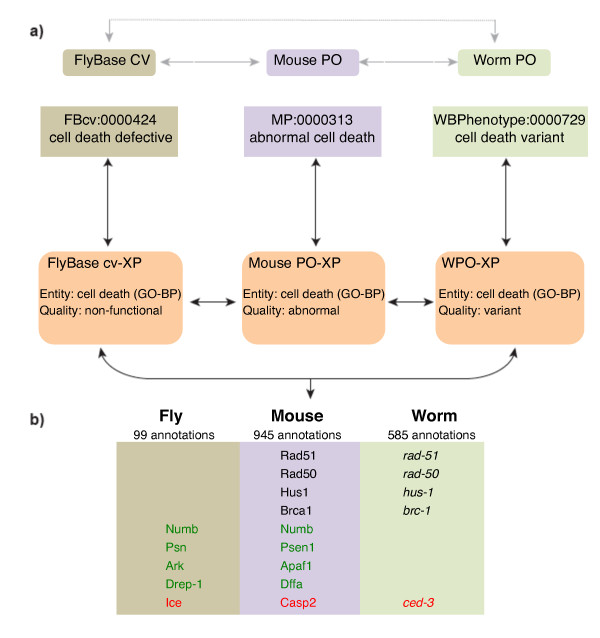
**Integrating phenotype ontologies across evolutionarily divergent species**. **(a) **Conceptual diagram depicting how multiple orthogonal phenotype ontologies (FlyBase Controlled Vocabulary, Mouse Phenotype Ontology, Worm Phenotype Ontology) can interact with each other via equivalence relationships (cross-products indicated by orange boxes). The example used here pertains to the 'cell death' process. XP stands for 'cross-product' and GO-BP stands for 'Gene Ontology Biological Process' **(b) **The table displays some of the phenotype annotations to genes relating to cell death anomalies in fly (*Drosophila melanogaster*), mouse (*Mus musculus*) and worm (*Caenorhabditis elegans*). Annotations were retrieved directly from their respective model organism databases (FlyBase, MGI, WormBase). Red font indicates conserved genes among all the depicted species. Green font shows conserved genes between *D. melanogaster *and *M. musculus*. Black font shows conserved genes between *C. elegans *and *M. musculus*.

To promote interoperability across different MODs and other biological databases we generated logical equivalence relationships (or cross-products) between process-oriented phenotype terms in the WPO and PATO-based EQ descriptions [17]. The availability of cross-products means that phenotype annotations to the pre-composed terms can be automatically converted to their corresponding EQ descriptions and vice versa. This conversion will facilitate communication between diverse organism phenotype ontologies and potentially improve data integration across communities (Figure 7a). We chose to generate equivalence mappings to the process-oriented phenotype terms because of their partial overlap with the Gene Ontology (GO), whose widespread adoption by distinct groups has played a crucial role in the integration of biological data [16]. Currently, the equivalence mappings we generated are not integrated into WormBase, but they can be accessed and viewed with an ontology editor (OBO-Edit, see Methods).

### Benefits of WPO-GO cross-products

The cross-products were generated manually so as to ensure logical coherence across ontologies and confirm biological validity. For instance the WormBase, FlyBase (http://flybase.org) and Mouse Genome Informatics (http://www.informatics.jax.org) databases contain 585, 99, and 945 'cell death' phenotype annotations, respectively (Figure 7b). Some of these phenotype associations appear to be conserved between their vertebrate and invertebrate gene orthologs, which suggests that mining genotype-phenotype connections from different organisms can lead to potentially useful predictions in other complex biological systems. In addition to constructing basic equivalence relations (based on semantic similarity), we were occasionally able to generate non-obvious yet biologically relevant Worm PO-GO cross-products by scrutinizing the literature and/or the definitions and synonyms of both WPO and GO-process terms, such as 'quiescence variant' and 'sleep' (Additional file 1) [40].

Another significant benefit of such equivalence mappings is the unmasking of cryptic terminologies that are often embedded within many individual phenotype terms or incorporated as synonyms. In other words, nematode 'species-centric' jargon (for instance, 'nose touch defective') becomes transparent to an outside researcher since the accurate equivalence mapping made to the parent term (namely, 'mechanosensation variant') would apply to the descendants as well (Additional file 2). This is a consequence of the is_a relationship, the more granular term (descendent) has general properties that it has inherited from its parent term [41].

Therefore the WPO, in conjunction with its validated equivalence mappings, can be utilized for cross-species queries and analyses of phenotype data derived from diverse resources. Although the simple pair-wise strategy can give incomplete results for complex phenotypes involving classes from more than one other ontology, this marks a good start towards constructing equivalences mappings in the WPO.

### Community driven evolution of the WPO

Our understanding of biology evolves over time. The underlying goal of community engagement is to respond to and provide the necessary channels to accommodate changes, while maintaining coherence and best practice methods during ontology development [42]. With this goal in mind, we have provided a means for user participation in the development and maintenance of the WPO.

**Figure 8:**
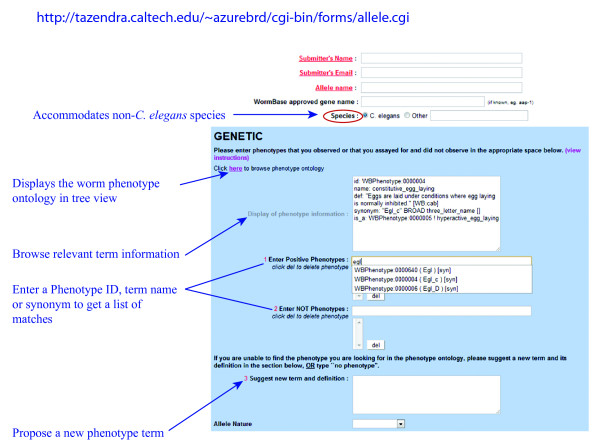
**Online user submission form for alleles**. The form allows users to browse the Worm Phenotype Ontology, assign phenotype terms to alleles or propose changes to the existing phenotype ontology. Submissions are reviewed prior to entry into the database. This form can also be accessed from the Allele data link on the WormBase Online Data Submission forms page at http://www.wormbase.org/db/curate/online_forms.

Users can directly interact with phenotype curators through the online allele submission form (which can be accessed at http://tazendra.caltech.edu/~azurebrd/cgi-bin/forms/allele.cgi, See Figure 8). The submission form gives users the option to browse the current ontology using term names, synonyms, or phenotype IDs (e.g. dumpy, Dpy, WBPhenotype:0000583). Users can also propose new phenotype terms or suggest revisions to the subject matter or placement of existing terms within the ontological tree. Such critical evaluation would ensure that the WPO would evolve alongside the nematode research we are committed to archiving.

## Discussion

We have developed the Worm Phenotype Ontology (WPO), a standardized syntax to classify and organize phenotype descriptions for *C. elegans *and other nematodes. The WPO will lead to consistency in phenotype description and an increase in curatorial efficiency at WormBase. The benefits of a controlled and structured phenotype vocabulary extends beyond WormBase users and will help integrate data from many different sources into a common body of knowledge, and facilitate data mining and comparisons across species. Hence, the WPO functions as a knowledge-based resource not only for *C. elegans *biologists, but also the larger biomedical, nematology, and bioinformatics communities.

### Improving data mining of phenotypic information

Search tools play an important role in the retrieval and display of bioinformatics data [43]. As stated above, WormBase has an ontology search that allows users to retrieve annotations to specific genes based on certain criteria, such as phenotypes. In addition, WormMart (http://wiki.wormbase.org/index.php/Data_mining:WormMart) serves as a more general data mining tool. We hope to extend the functionality of other existing WormBase tools to help in the dissemination of phenotype-based information, such as viewing phenotype annotations within the Genome Browser [44], which could potentially expedite positional cloning and gene mapping. Such a feature for the mouse genome has already been implemented by MGI [14]. An additional enhancement would be the incorporation of images and/or movies that represent a phenotype class.

In the future, WormBase also plans to implement the ability to conduct searches for allele, RNAi, transgene, or strain objects that do/do not exhibit a combination of defined phenotypes. This capability will allow users to further differentiate between allele phenotype classes. For example, one could distinguish between genes that perform a general excitatory role in neurotransmission versus genes that function only in specific behaviors/cell types. This capability will also be extended to performing combinatorial searches across ontologies (e.g., the anatomy ontology) in order to define genes that have been shown to be expressed, or to act, in specified cell types.

Given the expected increase of "other nematode" phenotypes in WormBase, we hope to provide search tools that render complex evolutionary questions amenable to computational analyses (Additional file 3). One example would be the ability to identify genes involved in the genetic pathways associated with hermaphroditic reproduction in different species that have undergone convergent evolution, as hermaphroditism has occurred independently in several clades during recent nematode evolution [45,46]. The basis for this type of analysis would be to examine phenotypes that are specifically associated with hermaphroditism. For example, *C. elegans *hermaphrodites require *fog-2 *to regulate the onset of spermatogenesis [47,48]. In contrast, the related species *C. briggsae *lacks *fog-2 *[49], but requires *she-1 *for execution of spermatogenesis in hermaphrodites [50]. A query for the 'spermatogenesis defective hermaphrodite' term would allow users to make the inference that both *fog-2 *and *she-1 *may act in the same pathway (Additional file 3). Additionally, such search tools would permit the identification of gene products that are co-opted for divergent functions across species. For instance, *gld-1 *promotes spermatogenesis in *C. elegans *hermaphrodites [51], whereas *C. briggsae gld-1 *appears to play the opposite role in germline sex determination (oogenesis) [49].

One strategy to quickly increase these cross-species data in WormBase is to curate RNAi experiments conducted with other nematodes such as *Heterorhabditis bacteriophora *[52]. The infrastructure for RNAi curation of other species exists at WormBase, the only caveat being that a stable reference genome is necessary to attach the sequence used in an RNAi experiment to a specific gene. In addition, we will rely on community input on mutant phenotypes in other species to help spearhead this effort.

Another opportunity for improving data synthesis is to improve the ability to store and retrieve phenotype information for syndromes in *C. elegans*. For example, 'Lag' (*lin-12 *and *glp-1*) phenotypes consist of 'nose twisted', 'no rectum', and 'excretory system development variant' phenotypes, a specific phenotype combination assigned to genes that are shared in processes controlled by the *lin-12 *and *glp-1 *Notch receptors in *C. elegans *[53]. One possibility is to develop a syndrome database model that captures the phenotype classes associated with each syndrome object. In the 'Lag' case mentioned above, we would create a 'Lag' syndrome object and if an allele, for example, displayed the 'nose twisted', 'no rectum', and 'excretory system development variant' phenotypes, it would automatically be associated with the 'Lag' syndrome. An extension of this approach, already being implemented by the Human Phenotype Ontology (HPO) group [54], would be to also create a search tool that looks not only at exact phenotype term matches for a specific syndrome, but is also able to traverse the pre-coordinated ontological structure for related terms and assign probability scores (significance threshold) for such results. For example, if an animal displays 'no rectum', 'excretory system development variant' and 'nose morphology variant' (the parent of 'nose twisted'), it would receive a lower but potentially significant confidence score for the 'Lag' syndrome because it does not contain the exact 'nose twisted' term, but has an exact match for the other two phenotypes.

### Intersections between orthogonal WPO, GO, and other ontologies

It is important to note the differences between annotation using the WPO compared to the GO. Whereas phenotype annotations are inextricably linked to the genetic background and conditions used to assay the phenotype, and these conditions are generally incorporated in the term's name or definition, manually assigned GO biological process annotations based upon mutant phenotypes (Inferred from Mutant Phenotype, or IMP) are meant to capture, as closely as possible, the biological processes directly affected by the gene. Thus, to assess an appropriate GO biological process annotation for a gene based upon a mutant phenotype, curators may consider additional information, such as the molecular identity of the gene product or, if known, the point in the process at which the gene product is believed to act. For example, a bHLH transcription factor that controls transcription of genes involved in muscle cell differentiation may exhibit a mutant phenotype of altered muscle contraction similar to that of mutations in genes encoding sarcomeric proteins. However, whereas both genes could be annotated to a 'muscle contraction variant' phenotype, the latter could be accurately annotated to the GO biological process term muscle contraction, while the former more accurately annotated to a term that reflects its role in regulation of muscle-specific gene transcription. The WPO and GO annotations would intersect at the level of a muscle-related process, but would differ in that the GO biological process annotation would capture the more specific roles of the individual gene products.

Logical equivalence relationships between ontologies also need to account for dependencies such as genetic background or assay and environmental conditions, and cross-species comparisons will be more meaningful when cross-products also incorporate this information in a standardized way using the various ontologies currently in development (OBI, the ontology for biomedical investigations; ChEBI, the chemical information ontology [55]; FIX, the ontology of physico-chemical methods and properties (http://www.obofoundry.org), etc.). Additional insight might be gained by these mappings and they will complement the synthesis of information that represents a GO annotation.

### Possible solutions to/challenges in creating cross-products to anatomy-based phenotypes

Much like the GO, the Worm Phenotype Ontology is continually undergoing active development. In addition to maintaining the existing ontological framework and enriching it with new phenotype classes, we are now in the process of generating cross-product ontologies for the non-process oriented phenotype classes (anatomy-based, chemical-based etc.). Besides facilitating phenotype comparisons across species, such equivalence relationships will help to build and maintain the WPO itself. As mentioned earlier, cross-product descriptions must be unambiguous and biologically sound in order to efficiently integrate phenotype data across various research organisms. However, the task of creating new cross-product ontologies, for example anatomy-based phenotypes, is accompanied by its own set of challenges.

For example, if we rely exclusively on the worm anatomy ontology developed in-house (http://www.obofoundry.org/cgi-bin/detail.cgi?id=worm_anatomy) for generating cross-products to anatomy-based phenotype terms, the utility of such equivalence mappings could potentially be restricted to the *C. elegans *community. One possible solution is to include UBERON [56], a uniform multi-species anatomy ontology, while generating cross-products to phenotypes involving certain generic anatomical entities (such as intestinal cell, striated muscle etc). UBERON has the added benefit of containing links to over 9,300 classes in other species-centric anatomical ontologies (besides the worm anatomy ontology), which could potentially simplify the retrieval of anatomy-based phenotypes across species. This is at best a partial solution because currently there is limited sharing of anatomical entities between UBERON and the worm anatomy ontology (40/6207 classes).

### Prediction of gene function and human disease models

Computational analyses of phenotype ontologies promotes the discovery of similarities between related phenotype abnormalities, which can subsequently be used for clinical diagnostic queries or as a basis for integrating phenotype and gene expression data sets to predict gene function [57,58], or other phenomena associated with complex human diseases.

The Worm PO-GO cross-product ontology (see above) is a valuable tool in terms of unmasking genes involved in fundamental processes that are shared among different species such as cell death, cell cycle etc. However, these equivalence relationships might not be as insightful when it comes to dissecting 'orthologous phenotypes' or phenotypes that arise from the disruption of a set of evolutionarily conserved genes that are differentially manifested across species. A recent study reported a method for identifying non-obvious equivalences between 'orthologous phenotypes' (phenologs) and human disease models [59]. These findings could be enriched to make predictions about gene networks involving phenologs by exploiting the hierarchical structure of multiple pre-coordinated phenotype ontologies. Namely, this would involve the recovery of genes that are annotated to phenotypes considered to be of a more specific subclass of the phenolog of interest, thereby aiding in the identification of additional candidate disease genes (Additional file 4). These results depend on the assumption that there can be a 1:1 equivalence mapping between the different phenotype classes of evolutionary distant species, for example human retinoblastoma eye cancer and ectopic vulvae in *C. elegans *[60,61].

At present, there is no individual platform that has the capacity to retrieve all existing mammalian and non-mammalian phenotypes that mirror well-documented human disorders, such as neurofibromatosis or Marfan syndrome. One plausible strategy would be to push towards more disease annotation; for instance, linking a specific mutation (and consequently its annotated phenotypes) to a human disease entry in OMIM [62]. This would presumably lead to the identification of other potential 'phenologs' thereby providing insights into evolutionary developmental biology and human disease states.

## Conclusions

Now that a framework for the Worm Phenotype Ontology is in place, further development and refinement of the ontology will occur in parallel with phenotype annotation; thus, evolution of the ontology will reflect the developing complexity with which phenotypes are described in nematodes. In addition to catering to the data mining needs of nematode biologists, one of our objectives is to make worm phenotypes accessible to the entire research community. In collaboration with other databases we ultimately envision the development of a web-based platform that integrates phenotype data, and data synthesis, across all MODs and other biological databases.

## Methods

We use OBO-Edit, under active development by the Gene Ontology Consortium (GOC) [63], for ontology development, refinement, and expansion. Ontology updates are committed to the WPO using CVS (Concurrent Versions System), served from a local PostgreSQL database, so that multiple curators can access and edit the ontology simultaneously. To facilitate internal collaboration on the development of the WPO, we have set up an internal web-based tool so that curators working on different phenotype-based data types (i.e., RNAi, allele, cell function, etc.) can request new terms. Along with the suggested term, curators suggest a definition and hierarchical placement within the ontology. The WormBase community can also request phenotype terms via an allele submission form (see Figure 8). A current version of the WPO is also available to the public at The OBO Foundry (Open Biological and Biomedical Ontologies) [64], which can be accessed here: http://caltech.wormbase.org/cvsweb/PhenOnt/ or from http://www.obofoundry.org/cgi-bin/detail.cgi?id=worm_phenotype.

OBO-Edit was also used to generate equivalence mappings for process-oriented phenotypes using the methodology described by Mungall and colleagues [17]. Each phenotype description consists of the following elements: E, the type of Gene Ontology (GO) process entity that is affected; Q, the quality borne by the entity. We refer to this collection of equivalence mappings as the Worm PO-GO-XP ontology (where XP stands for cross-product), which can be accessed at http://caltech.wormbase.org/cvsweb/PhenOnt/.

Allele and transgene phenotypes were initially curated via Phenote (http://www.phenote.org), a software application that facilitates phenotype annotation using ontologies. As of October 2009, we switched to a web-based ontology annotation tool (developed in-house; details to be described elsewhere) for the curation of alleles, transgenes and strains. RNAi sequence mapping tools were developed in-house.

## Authors' contributions

CAB and PWS initiated this project. CAB, GS, JSF and KY further developed the WPO, namely creating, defining and placing terms in the ontology. JSF and GS generated and maintained the WPO-GO XP ontology. GS, JSF and CAB wrote the paper with valuable discussions and critical contributions at all stages of the project from KY and PWS. All authors read and approved the final manuscript.

## Supplementary Material

Additional file 1 **Figure S1. Construction of non-obvious yet biologically relevant equivalence mappings**. **(a) **Equivalence relationship between the 'quiescence variant' phenotype class and its corresponding EQ description. E = GO term 'sleep' and Q = PATO term 'variant'. **(b) **This table displays some of the phenotype annotations to genes relating to sleep anomalies in fly (*Drosophila melanogaster*), mouse (*Mus musculus*) and worm (*Caenorhabditis elegans*). Annotations were retrieved directly from their respective model organism databases (FlyBase, MGI, WormBase). Red font indicates conserved genes among all the depicted species. Green font shows conserved genes between *D. melanogaster *and *C. elegans*. Black font shows conserved genes between *D. melanogaster *and *M. musculus*. Click here for file

Additional file 2 **Figure S2. Cross-products assigned to the 'mechanosensation variant' class apply to all of the granular subclasses as well (such as 'nose touch defective')**. Shown on left is the 'mechanosensation variant' term in the context of the WPO. Its cross-products are indicated by dashed lines in OBO-Edit on the Cross Products Table Here 'Intersection Genus' represents 'Quality' and the 'Discriminating Relationships' represent 'Entity'. Also shown (blue arrow) is the term definition of one of the subclasses ('nose touch defective'). The cross product to the parent applies to this child term as well. Click here for file

Additional file 3 **Figure S3. Potential application of mining phenotypic data for multiple nematode species**. Shown is a theoretical table generated by querying WormBase for 'spermatogenesis defective hermaphrodite'. The results include genes (annotated to this phenotype term) along with their corresponding species and reference. Green font depicts genes with different molecular functions that are both involved in *tra-2 *repression to promote XX spermatogenesis (convergence) and red font depicts a gene that has been co-opted for an alternate function in *C. briggsae*. Click here for file

Additional file 4 **Figure S4. Exploiting the hierarchy of pre-coordinated phenotype ontologies to acquire data on gene networks involving 'orthologous phenotypes' and their relationship to human disease**. The example illustrated is the Notch/Delta family (pink oval), its connection to human disease (green oval) and a corresponding phenotype connection to the mouse and worm phenotype ontologies ('abnormal hematopoiesis' and 'germline proliferation variant' are the respective terms). Red font points to direct associations with the parent terms and blue fonts bracket the connections to the descendent terms. Click here for file
