# Supplementary material for: Worm Phenotype Ontology: Integrating phenotype data within and beyond the C. elegans community
Source: BMC Bioinformatics. 2011 Jan 24;12:32. doi: 10.1186/1471-2105-12-32 (PMC3039574; doi:10.1186/1471-2105-12-32)
Supplement: Additional file 1 — Figure S1. Construction of non-obvious yet biologically relevant equivalence mappings. (a) Equivalence relationship between the 'quiescence variant' phenotype class and its corresponding EQ description. E = GO term 'sleep' and Q = PATO term 'variant'. (b) This table displays some of the phenotype annotations to genes relating to sleep anomalies in fly (Drosophila melanogaster), mouse (Mus musculus) and worm (Caenorhabditis elegans). Annotations were retrieved directly from their respective model organism databases (FlyBase, MGI, WormBase). Red font indicates conserved genes among all the depicted species. Green font shows conserved genes between D. melanogaster and C. elegans. Black font shows conserved genes between D. melanogaster and M. musculus. [file 1471-2105-12-32-S1.PDF]

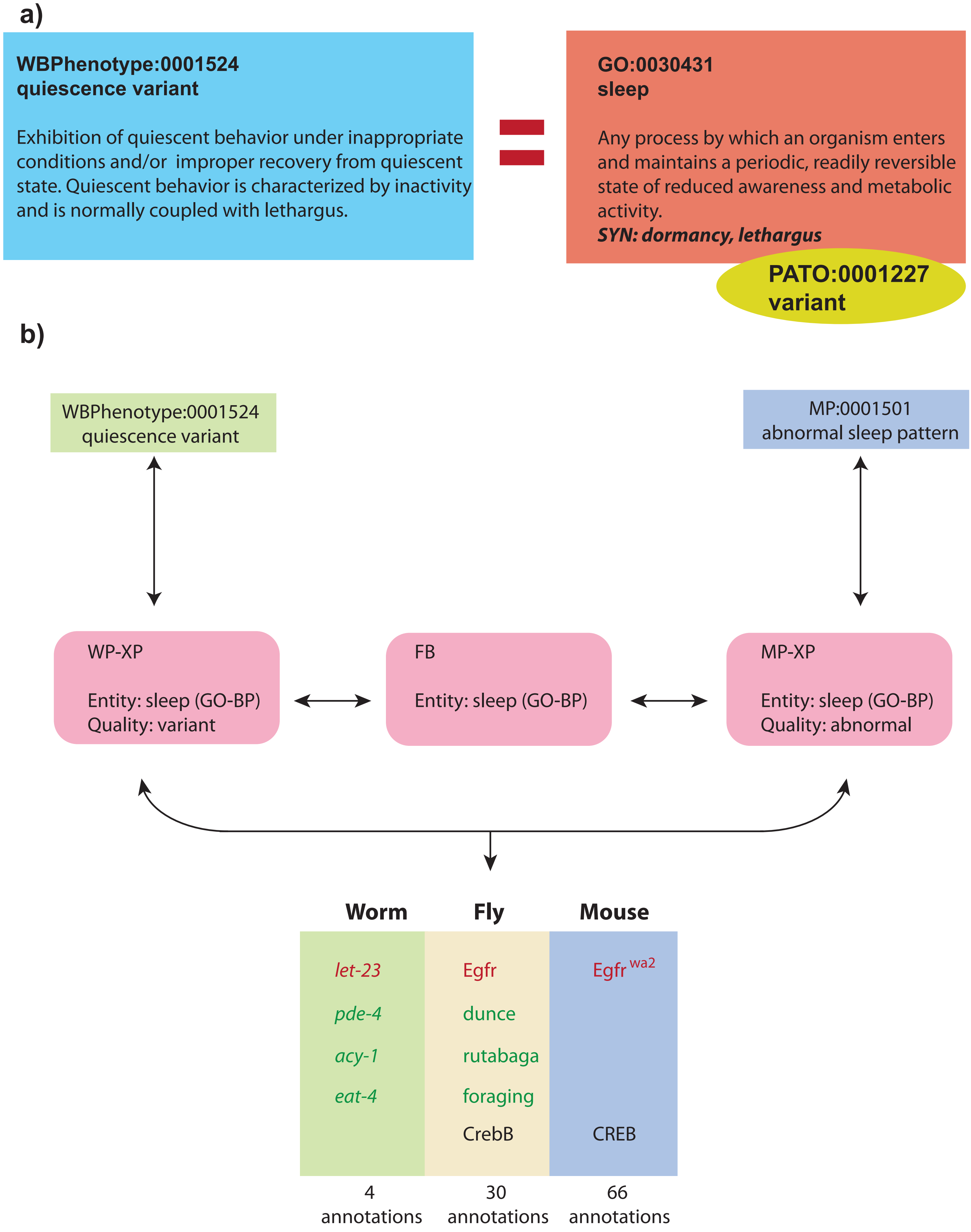
a)
WBPhenotype:0001524
quiescence variant
Exhibition of quiescent behavior under inappropriate conditions and/or improper recovery from quiescent state. Quiescent behavior is characterized by inactivity and is normally coupled with lethargus.
GO:0030431
sleep
Any process by which an organism enters and maintains a periodic, readily reversible state of reduced awareness and metabolic activity.
SYN: dormancy, lethargus
PATO:0001227
variant
b)
WBPhenotype:0001524
quiescence variant
MP:0001501
abnormal sleep pattern
WP-XP
Entity: sleep (GO-BP)
Quality: variant
FB
Entity: sleep (GO-BP)
MP-XP
Entity: sleep (GO-BP)
Quality: abnormal
Worm
Fly
Mouse
let-23
Egfr
Egfr wa2
pde-4
dunce
acy-1
rutabaga
eat-4
foraging
CrebB
CREB
4
annotations
30
annotations
66
annotations
