# Supplementary material for: Worm Phenotype Ontology: Integrating phenotype data within and beyond the C. elegans community
Source: BMC Bioinformatics. 2011 Jan 24;12:32. doi: 10.1186/1471-2105-12-32 (PMC3039574; doi:10.1186/1471-2105-12-32)
Supplement: Additional file 2 — Figure S2. Cross-products assigned to the 'mechanosensation variant' class apply to all of the granular subclasses as well (such as 'nose touch defective'). Shown on left is the 'mechanosensation variant' term in the context of the WPO. Its cross-products are indicated by dashed lines in OBO-Edit on the Cross Products Table Here 'Intersection Genus' represents 'Quality' and the 'Discriminating Relationships' represent 'Entity'. Also shown (blue arrow) is the term definition of one of the subclasses ('nose touch defective'). The cross product to the parent applies to this child term as well. [file 1471-2105-12-32-S2.PDF]

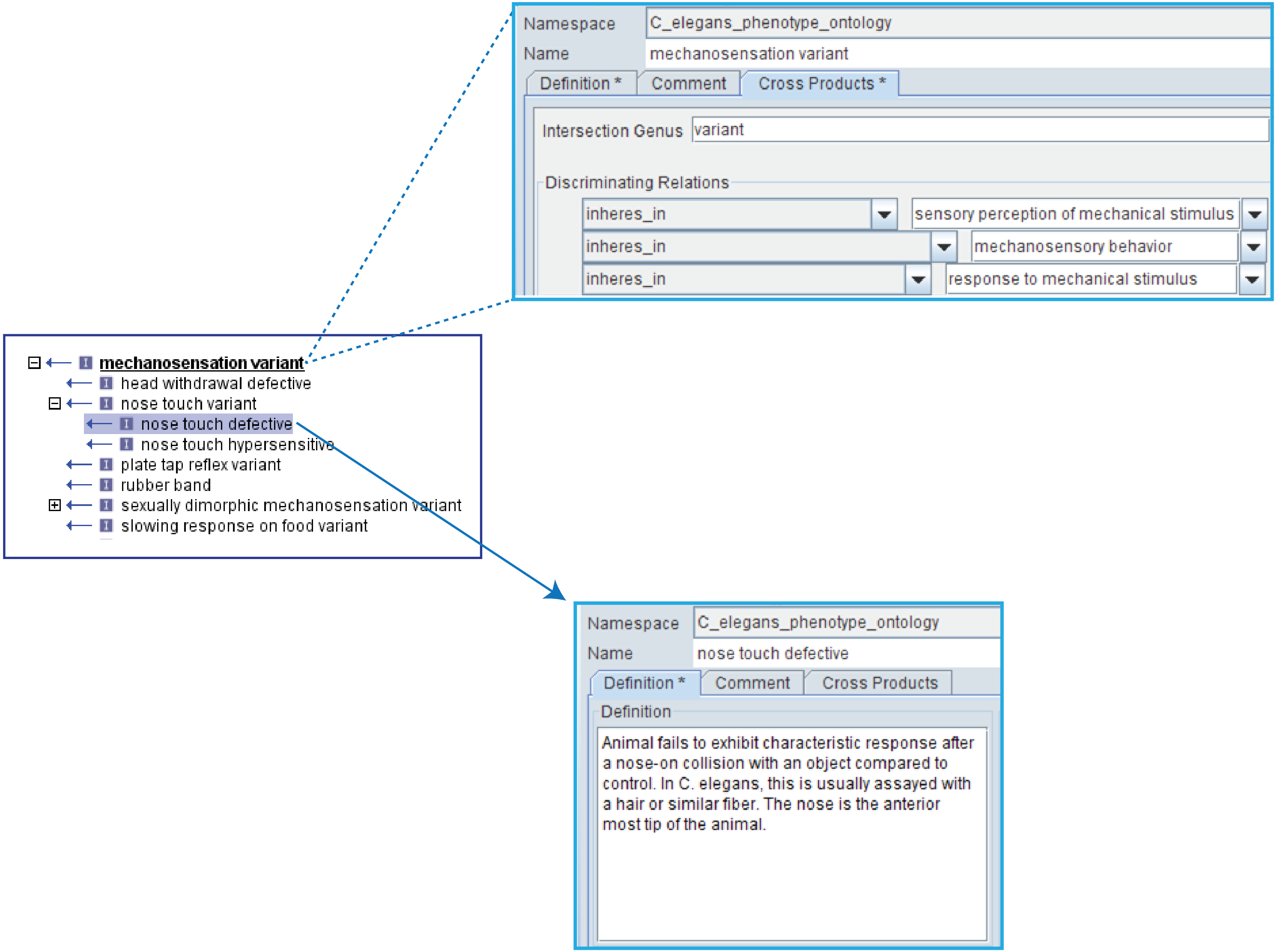
Namespace
C_elegans_phenotype_ontology
Name
mechanosensation variant
Definition *
Comment
Cross Products *
Intersection Genus
variant
Discriminating Relations
inheres_in
sensory perception of mechanical stimulus
inheres_in
mechanosensory behavior
inheres_in
response to mechanical stimulus
mechanosensation variant
head withdrawal defective
nose touch variant
nose touch defective
nose touch hypersensitive
plate tap reflex variant
rubber band
sexually dimorphic mechanosensation variant
slowing response on food variant
Namespace
C_elegans_phenotype_ontology
Name
nose touch defective
Definition *
Comment
Cross Products
Definition
Animal fails to exhibit characteristic response after a nose-on collision with an object compared to control. In C. elegans, this is usually assayed with a hair or similar fiber. The nose is the anterior most tip of the animal.
