# Supplementary material for: Worm Phenotype Ontology: Integrating phenotype data within and beyond the C. elegans community
Source: BMC Bioinformatics. 2011 Jan 24;12:32. doi: 10.1186/1471-2105-12-32 (PMC3039574; doi:10.1186/1471-2105-12-32)
Supplement: Additional file 3 — Figure S3. Potential application of mining phenotypic data for multiple nematode species. Shown is a theoretical table generated by querying WormBase for 'spermatogenesis defective hermaphrodite'. The results include genes (annotated to this phenotype term) along with their corresponding species and reference. Green font depicts genes with different molecular functions that are both involved in tra-2 repression to promote XX spermatogenesis (convergence) and red font depicts a gene that has been co-opted for an alternate function in C. briggsae. [file 1471-2105-12-32-S3.PDF]

Convergence

Phenotype	Gene	Species	Reference
Spermatogenesis defective hermaphrodite	*fog-2*	*C. elegans*	Schedl et al, 1988
Spermatogenesis defective hermaphrodite	*she-1*	*C. briggsae*	Guo et al, 2009
Spermatogenesis defective hermaphrodite	*spe-8*	*C. elegans*	L'Hernault et al, 1988
Spermatogenesis defective hermaphrodite	*spe-12*	*C. elegans*	L'Hernault et al, 1988
Spermatogenesis defective hermaphrodite	*prg-2*	*C. elegans*	Das et al, 2008
Spermatogenesis defective hermaphrodite	*prg-1*	*C. elegans*	Das et al, 2008
Spermatogenesis defective hermaphrodite	*gld-1*	*C. elegans*	Francis et al, 1995

Functional Divergence

Phenotype	Gene	Species	Reference
Masculinization of germline	*gld-1*	*C. briggsae*	Nayak et al, 2004
