# Supplementary material for: Worm Phenotype Ontology: Integrating phenotype data within and beyond the C. elegans community
Source: BMC Bioinformatics. 2011 Jan 24;12:32. doi: 10.1186/1471-2105-12-32 (PMC3039574; doi:10.1186/1471-2105-12-32)
Supplement: Additional file 4 — Figure S4. Exploiting the hierarchy of pre-coordinated phenotype ontologies to acquire data on gene networks involving 'orthologous phenotypes' and their relationship to human disease. The example illustrated is the Notch/Delta family (pink oval), its connection to human disease (green oval) and a corresponding phenotype connection to the mouse and worm phenotype ontologies ('abnormal hematopoiesis' and 'germline proliferation variant' are the respective terms). Red font points to direct associations with the parent terms and blue fonts bracket the connections to the descendent terms. [file 1471-2105-12-32-S4.PDF]

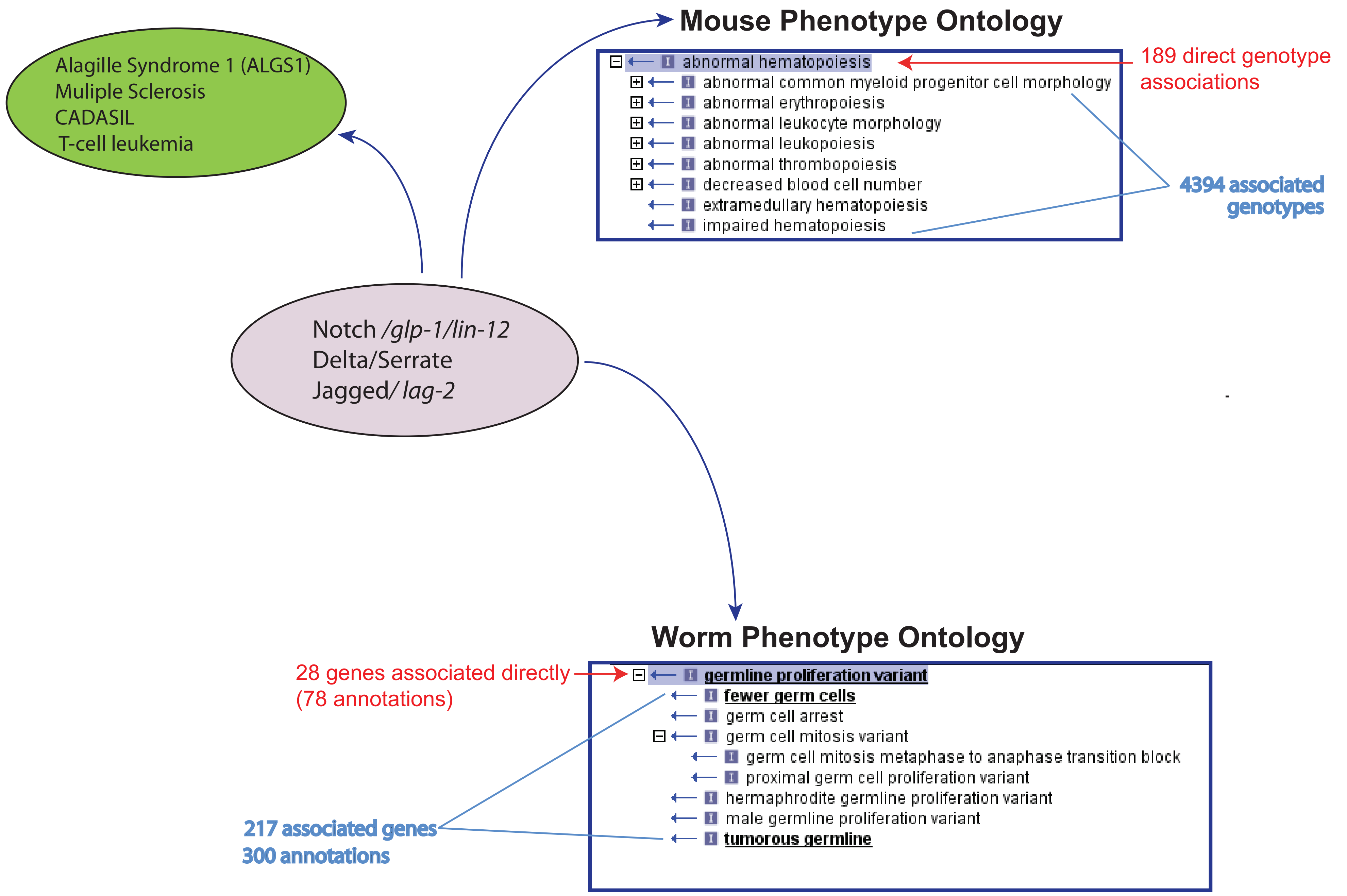

Mouse Phenotype Ontology
abnormal hematopoiesis
abnormal common myeloid progenitor cell morphology
abnormal erythropoiesis
abnormal leukocyte morphology
abnormal leukopoiesis
abnormal thrombopoiesis
decreased blood cell number
extramedullary hematopoiesis
impaired hematopoiesis
189 direct genotype associations
4394 associated genotypes
Alagille Syndrome 1 (ALGS1)
Muliple Sclerosis
CADASIL
T-cell leukemia
Notch /*glp-1/lin-12*
Delta/Serrate
Jagged/ *lag-2*
Worm Phenotype Ontology
germline proliferation variant
fewer germ cells
germ cell arrest
germ cell mitosis variant
germ cell mitosis metaphase to anaphase transition block
proximal germ cell proliferation variant
hermaphrodite germline proliferation variant
male germline proliferation variant
tumorous germline
28 genes associated directly
(78 annotations)
217 associated genes
300 annotations
